# The Enhancement of Antimicrobial Photodynamic Therapy of *Escherichia Coli* by a Functionalized Combination of Photosensitizers: In Vitro Examination of Single Cells by Quantitative Phase Imaging

**DOI:** 10.3390/ijms23116137

**Published:** 2022-05-30

**Authors:** Aleksandra Pietrowska, Iwona Hołowacz, Agnieszka Ulatowska-Jarża, Mateusz Guźniczak, Anna K. Matczuk, Alina Wieliczko, Mirela Wolf-Baca, Igor Buzalewicz

**Affiliations:** 1 Department of Biomedical Engineering, Faculty of Fundamental Problems of Technology, Wroclaw University of Science and Technology, 27 Wybrzeże S. Wyspiańskiego St., 50-370 Wrocław, Poland; aleksandra.pietrowska@pwr.edu.pl (A.P.); iwona.holowacz@pwr.edu.pl (I.H.); agnieszka.ulatowska-jarza@pwr.edu.pl (A.U.-J.); 249986@student.pwr.edu.pl (M.G.); 2 Department of Pathology, Division of Microbiology, Faculty of Veterinary Medicine, Wrocław University of Environmental and Life Sciences, 31 C.K. Norwida St., 51-375 Wrocław, Poland; anna.matczuk@upwr.edu.pl; 3 Department of Epizootiology and Veterinary Administration with Clinic of Infectious Diseases, Wrocław University of Environmental and Life Sciences, 45 Grunwaldzki Square, 50-366 Wrocław, Poland; alina.wieliczko@upwr.edu.pl; 4 Department of Environmental Protection Engineering, Faculty of Environmental Engineering, Wroclaw University of Science and Technology, 27 Wybrzeże S. Wyspiańskiego St., 50-370 Wrocław, Poland; mirela.wolf-baca@pwr.edu.pl

**Keywords:** antimicrobial photodynamic therapy, photodynamic inactivation, digital holotomography, quantitative phase imaging, Chlorin e6, Pheophorbide a, *E. coli*

## Abstract

The prevention of biofilm formation is crucial for the limitation of bacterial infections typically associated with postoperative infections, complications in bedridden patients, and a short-term prognosis in affected cancer patients or mechanically ventilated patients. Antimicrobial photodynamic therapy (aPDT) emerges as a promising alternative for the prevention of infections due to the inability of bacteria to become resistant to aPDT inactivation processes. The aim of this study was to demonstrate the use of a functionalized combination of Chlorin e6 and Pheophorbide as a new approach to more effective aPDT by increasing the accumulation of photosensitizers (PSs) within *Escherichia coli* cells. The accumulation of PSs and changes in the dry mass density of single-cell bacteria before and after aPDT treatment were investigated by digital holotomography (DHT) using the refractive index as an imaging contrast for 3D label-free live bacteria cell imaging. The results confirmed that DHT can be used in complex examination of the cell–photosensitizer interaction and characterization of the efficiency of aPDT. Furthermore, the use of Pheophorbide a as an efflux pomp inhibitor in combination with Chlorin e6 increases photosensitizers accumulation within *E. coli* and overcomes the limited penetration of Gram-negative cells by anionic and neutral photosensitizers.

## 1. Introduction

Biofilms are responsible for up to 80% of all causes of chronic and recurrent bacterial infections in humans [1]. The presence of biofilms has been confirmed on more than 90% of dry healthcare surfaces in intensive care units [2]. Currently, the presence of antibiotic-resistant bacteria and the infections caused by them are a huge threat to public health. This condition is mainly due to the overuse of antibiotics [3]. Microbes are capable of attaching to surfaces and forming a biofilm. Mature biofilms are up to 1000 times less susceptible to dehydration, phagocytosis, metal toxicity, acid exposure, antibiotics, and biocides than bacteria in planktonic culture [4]. Biofilms are generally associated with wet and damp surfaces and may be difficult to remove with the current decontamination process [5]. These complex three-dimensional structures may comprise the same or different bacteria species. Microbial cells constitute 2–5% of the biofilm, other major components are water (up to 97%), polysaccharides, proteins and enzymes, DNA, and RNA. Approximately 80% of surgical site infections (SSIs) are caused by biofilm-forming bacteria. *E. coli* belongs to the group of microorganisms related to this common postoperative occurrence [6]. In particular, Gram-negative bacteria are considered more pathogenic, since Gram-positive bacteria are more susceptible to antibiotics because they do not contain an outer membrane. The main difference between these types of bacteria is that Gram-positive bacteria have a thick cell wall made of peptidoglycans, with teichoic acid found in some bacteria, while Gram-negative bacteria have a relatively thin wall without said acid. Additionally, lipopolysaccharide chains are a characteristic feature of the outer membrane of Gram-negative bacteria [7].

The mechanism of antimicrobial photodynamic therapy (aPDT) is based on the selective accumulation by the bacterial cell of a nontoxic dye called photosensitizer (PS), its activation by light of a specific wavelength in the visible range in the presence of oxygen resulting in the formation of highly harmful and reactive oxygen species [8]. In the context of the worldwide rise of bacterial antibiotics, photodynamic therapy may be a beneficial alternative to the conventional treatment method [9]. Moreover, photodynamic therapy is considered to be a novel strategy to control biofilm-associated SSIs [6]. Over 50% of the bacteria on the NIAID list of critical pathogens have been treated with this strategy at least one time [10]. Its main advantage is the inability of bacteria to become resistant to inactivation processes [11,12,13]. It is commonly assumed that the bacteria inactivation from aPDT is resulting by either cytoplasmic membrane or DNA damage, depending on place of PS accumulation. In [14], three hypotheses on damage to bacterial cells were described:

- The photosensitizer is in close proximity to the bacteria but is not related in any way. In this way, damage to the structural elements of the bacteria is limited.
- The photosensitizer binds to the bacteria as a result of electrostatic, van der Waals, or hydrogen bonding interactions. Then the chance of damage to even the outer membrane increases.
- The photosensitizer penetrates the bacteria in several steps. The first is membrane binding, then active transport or diffusion into the cytoplasm. Such a location even allows DNA damage.

Recently, the main objective of aPDT is to increase the efficiency of bacteria cells by PSs, which depends not only on the structure and chemophysical properties of PSs, but also on the structure of the bacterial cell, mainly the structure of the call wall. Not all available photosensitizers can be used for aPDT. There are many properties of photosensitizers that affect their accumulation in cells and the efficiency of aPDT, including chemical purity, stability, low price, highest absorption in the visible range, high quantum efficiency, and, most importantly, it cannot be toxic in the dark [15]. New compounds that could be used in photodynamic therapy are constantly searched for. One of the promising prospects is the use of metallodrugs as photosensitizers. Ru(II) polypyridyl complexes are characterized by a variety of electronic configurations of the metal in the excited state accessible with near-infrared and visible light. Furthermore, configurations may be tunable by ligand tailoring [16]. Ruthenium-based metal complexes and ruthenium nanoparticles have also found application in photodynamic antimicrobial studies [17,18].

However, the mechanisms of cell penetration by PS also differ significantly in the context of planktonic cells or biofilms. In the case of the biofilm structure, its penetration by PS is limited by extracellular polymeric substances [19]. In the 1990s, a difference in the effectiveness of photodynamic therapy was observed in the case of Gram-positive and Gram-negative bacteria. Anionic and neutral photosensitizers bind to Gram-positive bacteria and therapies are highly effective, but in the case of Gram-negatives, they do not bring the expected results. It is related to the structure of bacteria. Gram-positive cells have a cytoplasmic membrane with porous peptidoglycan and lipoteichoic acid, which allows noncharged molecules to bind to them. Negatively charged lipopolysaccharide molecules weaken the possibility of adsorption of neutral photosensitizers on the membrane and repel anionic photosensitizers as a result of electrostatic interactions. Therefore, they are not inactivated [20]. For these reasons, numerous studies are being carried out in order to develop new PSs that will be able to accumulate more effectively in Gram-negative bacteria cells.

The cell wall of Gram-negative bacteria is characterized by low permeability, so only selected photosensitizers have the ability to penetrate the cell wall. This is related to the electric charge of the PS and the cell membrane. Cationic porphyrin can be adsorbed on the anionic outer membrane; amphiphilic porphyrin has the ability to interact with parts of the outer and inner membranes. Ultimately, the compound has the potential to pass through both membranes and into bacteria [21]. In addition, the penetration of the photosensitizer into bacteria may be related to a specific uptake pathway (self-promotion mechanism). The cationic photosensitizer binds to lipopolysaccharides, which displaces Ca^2+^ and Mg^2+^ cations and creates gaps in the outer membrane. It is with their help that the cationic compound is incorporated into the bacteria. It was also confirmed that increasing the amount of Mg^2+^ cations reduces photosensitizer uptake [19]. Therefore, control of the mechanism of action of membrane efflux pumps required for the extrusion of substrates from the cellular interior to the surrounding medium can also affect the accumulation of PS.

The main objective of this study was to demonstrate the increase in PS accumulation within bacteria single-cells by using the functionalized combination of anionic Chlorin e6 (Ce6) and cationic Pheophorbide a (Pheo) PS instead of each individual. As will be demonstrated, Pheo exhibits a lower accumulation within the cell than Ce6, but, being the efflux pump inhibitor (EPI) [22,23] it can be used in combination with anionic Ce6 to improve Ce6 accumulation within the bacteria cell and consequently also to perform a more effective aPDT. The examination was carried out on *Escherichia coli* bacteria belonging to Gram-negative bacteria, as its cell membrane restricts cell penetration by anionic and neutral photosensitizers [24]. The accumulation of PSs was investigated by digital holotomography (DHT) using the refractive index (RI) as an imaging contrast for 3D label-free live bacteria cell imaging, since RI is related to the optical density of cells related to their mass and the cellular concentration of individual components [25]. As confirmed, the accumulation of photosensitizers within bacteria cells leads to changes in cell density and, consequently, to changes in RI values [25,26]. In a recent study, it was demonstrated that DHT is able to indicate, in a quantitative, nondestructive, and label-free manner, the difference in PS accumulation inside the bacteria cells with the use of each PS individually and in combination. Furthermore, DHT-derived RI data will be demonstrated to provide insight into differences in the dry mass density of individual cells related with the effectiveness of aPDT treatment. The results suggest that the use of functionalized photosensitizer mixtures can contribute to an increase in the efficiency of bacterial cell penetration and can create new perspectives for more efficient aPDT in the case of Gram-negative bacteria species, which was confirmed based on *E. coli* examination.

## 2. Results and Discussions

### 2.1. Photosensitizers’ Spectroscopic Properties

**Figure 1:**
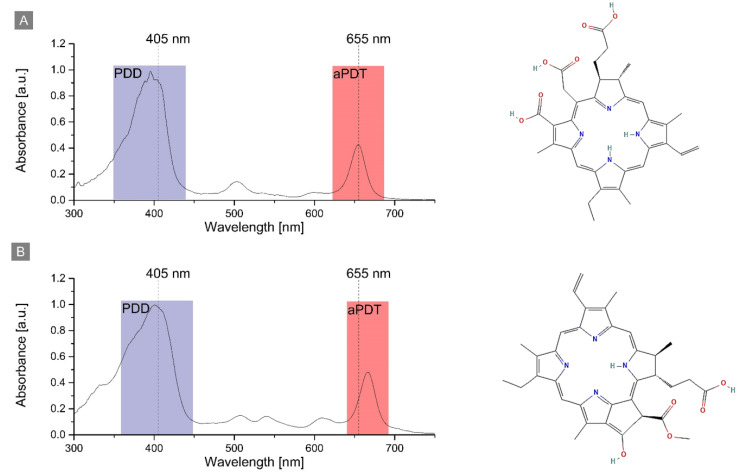
The structure and absorption of (**A**) Chlorin e6 and (**B**) Pheophorbide a in PBS buffer with indication of the used wavelengths for PDD and aPDT.

The representative absorption spectra of the PS are presented in Figure 1. For Ce6 and Pheo, the relevant spectral bands were used for antimicrobial photodynamic therapy (aPDT) of cells, and the photodynamic diagnosis (PDD). PDD was used for evaluation of the efficiency of cells penetration by PS, since the intensity of the photosensitizer fluorescence is directly correlated with its concentration and can be examined by confocal fluorescence microscopy. 

The absorption spectrum of Ce6 and Pheo shows a strong band at 397 nm and 402 nm, respectively. This allowed the visualization of PS accumulation inside the cell by fluorescence imaging with a 405 nm laser. The second absorption band was used for aPDT treatment and photosensitizer excitation by the laser diode (λ = 655 nm). The PS photoexcitation wavelength used for aPDT was the same in the case of all photosensitizers: Ce6, Pheo, and Ce6 + Pheo.

### 2.2. Study of the Photosensitizers Interaction with Bacteria by Confocal Microscopy

**Figure 2:**
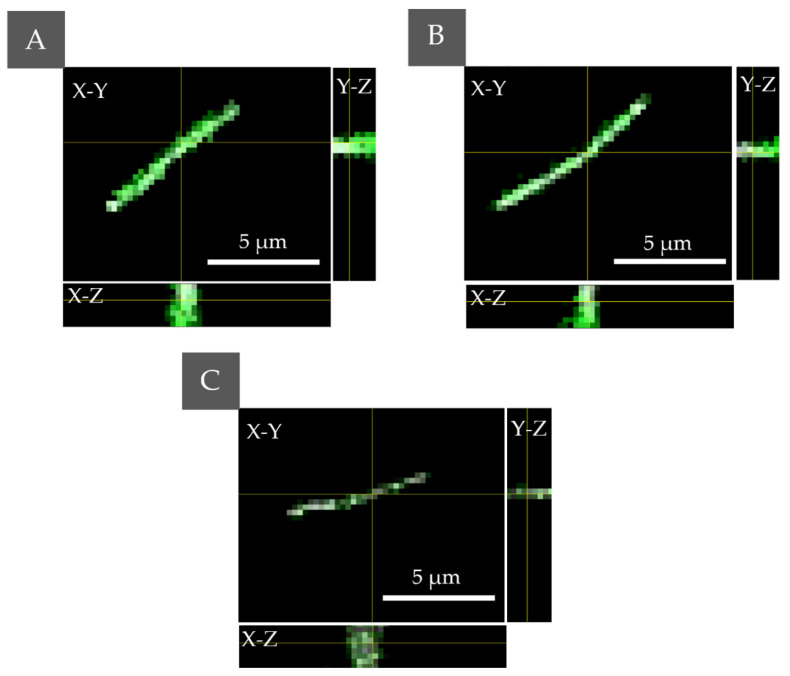
The PS localization performed by fluorescence confocal microscopy for *E. coli* with accumulated (**A**) Ce6 + Pheo, (**B**) Ce6, (**C**) Pheo—the combined fluorescence–DIC images with axial cross sections: X–Z/Y–Z (the yellow lines indicate the cross section through a cell).

Examination of the photosensitizer interaction with cells was performed by scanning fluorescence confocal microscopy. *E. coli* cells incubated for 24 h with PS were examined to prove whether PSs were accumulated on the cell’s wall or inside cell. The colocalization of fluorescence and bacteria cell is in this case used as an indicator of PS’s accumulation inside the bacteria cell. As can be seen, PS fluorescence was observed in all samples with added PS solution, which confirms the presence of the nonaggregated form of PS. Selected slices from the Z-scan are shown in Figure 2. 

The results confirm the ability of all the used PSs to penetrate the bacteria cells, but with different efficiency. Pheo belongs to the cationic PS and its transport is mediated by electrostatic interactions and self-promoted uptake pathways [27]. Anionic PSs (Ce6) can be mediated into bacterial cells through a combination of electrostatic charge interaction and protein transporters. The lowest fluorescence in the case of Pheo indicates that cell penetration by this PS was significantly lower than in the case of Ce6 or combination of PS. The highest fluorescence signal and accumulation inside the cell were observed in case of the combination of PSs. Moreover, the confocal microscopic examination did not indicate changes in the morphology of bacteria cells under 405 nm radiation in the DIC mode, which corresponds also to the lack of changes in the cell density or dry mass density due to the possible photoinactivation.

### 2.3. Bacterial Cell Penetration by Photosensitizers

#### 2.3.1. The Examination of the Possible PSs Accumulation Inside the Bacteria Cells by DHT

**Figure 3:**
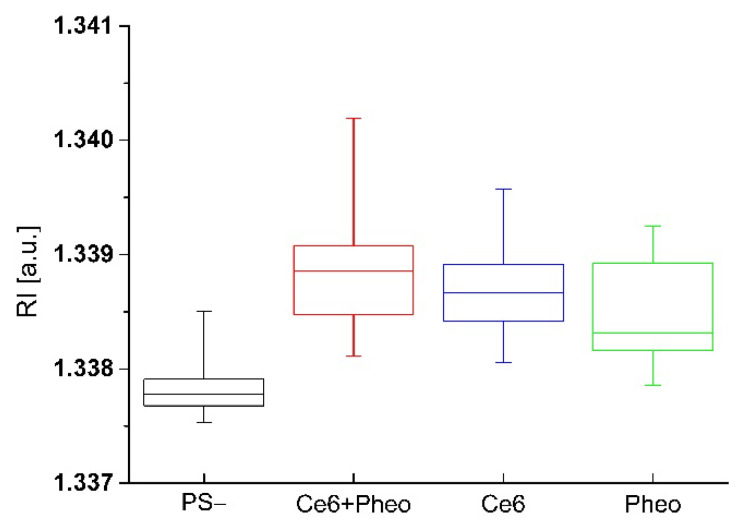
The change of RI values of cells with and without PS after 24 h incubation.

The impact of accumulation of PSs on the cell wall or penetration of the cell interior and aPDT on the averaged RI value of the cells was analyzed. Variation of local RI values is associated with the examined cell physiology but also with local fluctuations of its density, dependent on the chemical composition and external factors. In our approach, the increase in the average RI value of bacteria cells was related to the increase in cell density caused by the accumulation of PS. The accumulation of PS in the cell interior or cell membranes leads to an increase in RI value but can also be associated with physiological processes such as cell division [26]. To obtain a representative set of data for this analysis, 20 tomograms (3D-RI) of each group were examined. For all examined photosensitizers, an increase in the averaged RI values of cells was observed (see Figure 3). The averaged RI value was higher in the case of cells with PSs rather than the control sample without PS, what suggest that such significant increase in RI value has to be related with the accumulation of PS and not cell division. Furthermore, the greatest difference in the averaged RI value was observed for Ce6 + Pheo, indicating a more effective cell penetration by such a combination of PSs. This process may be caused by the use of Pheo as an efflux pump inhibitor [22,23], which contributed to a more effective accumulation of photosensitizers within bacteria cells. Differences in medians between PS− and Pheo + Ce6, Ce6, Pheo were 0.00108, 0.00088, 0.00053, respectively.

**Figure 4:**
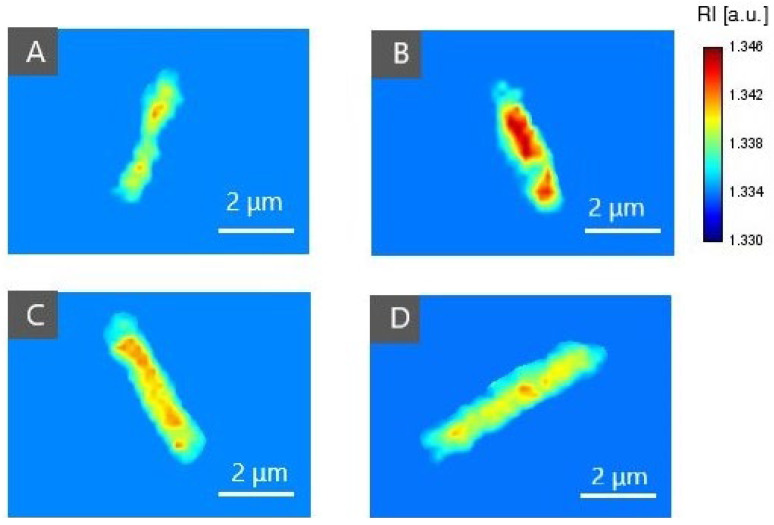
The representative 2D-RI maps of *E. coli* cells incubated without PS (**A**) and incubated with Ce6 + Pheo (**B**), Ce6 (**C**), or Pheo (**D**).

For confirmation of PS (Ce6 + Pheo, Ce6, Pheo) inside bacteria cells after 24 h of incubation, 2D-RI maps were used to colocalize the region of cells with the highest RI values. Each 2D-RI map represents the lateral RI distribution in the plane corresponding to the maximum of a single bacteria cell. The results are shown in Figure 4. As a reference, single *E. coli* cells not incubated with any photosensitizers were used (see Figure 4A). For all examined cells incubated with photosensitizers, an increase in RI values was observed in the intracellular region. Representative 2D-RI maps confirm that the highest RI values were obtained for the combination of both photosensitizers Ce6 + Pheo (see Figure 4B), which is related to the increase in density inside the cells caused by accumulation of PSs. The DHT results correlate with the results of the confocal microscopic examination.

To determine whether the observed changes in the averaged RI value of cells caused by the interaction of PS with cells were statistically significant, a one-way ANOVA was performed. The normality assumption of the average RI values of cells was confirmed by the Kolmogorov–Smirnov test at the 5% significance level. The ANOVA results are shown in Table 1. The estimated *p*-value for the F-statistic is significantly smaller (6.827 × 10^−11^) than the significance level (0.05), which means that the test rejected the null hypothesis that all means of the group were equal.

Furthermore, the variability between groups was higher than the variability within the groups. It indicates that the accumulation of the PSs significantly influenced the averaged RI value of the bacterial cells. Therefore, the analysis performed showed that the 3D-RI data provided by DHT could be used to visualize local density changes related to the accumulation of PS inside the cells.

#### 2.3.2. The Examination of Antibacterial Efficiency of PSs by DHT

The examinations were performed before and 24 h after the irradiation of the bacterial cells. The 3D-RI values of the bacteria cells (the averaged 3D-RI values of the voxels of the cell) were determined by averaging the RI values of the pixels of the region occupied by bacteria cells for each of the 2D-RI maps (cross sections) through the cell. It should be noted that bacteria cells are an object in which the spatial distribution of the refractive index is related not only to the specific concentration of chemical components, such as proteins or sugars, but also made of the structures such as DNA material, cell walls/membranes, flagella, pili, etc. However, the determination of the minimal local changes of the RI values that indicate these structures is limited by the lateral/axial resolution of the DHT which was already indicated in [28,29]. 

**Figure 5:**
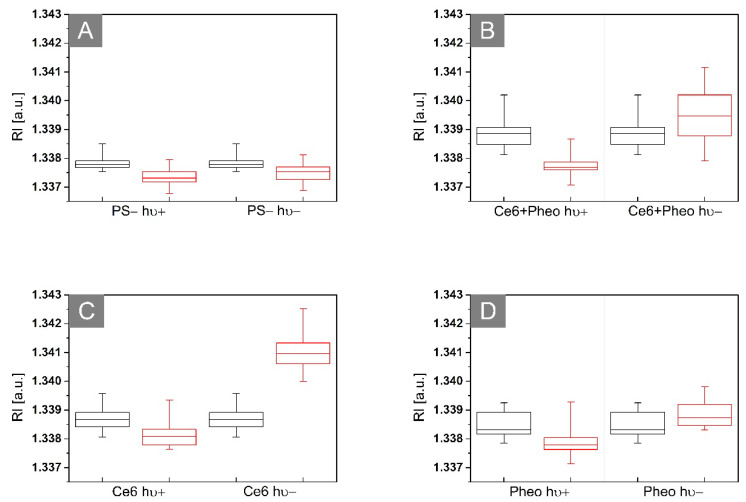
Representative box plots of 3D-RI values of *E. coli* cells 24 h (black boxes) and 48 h (red boxes) after incubation without PS (**A**) or with Ce6 + Pheo (**B**), Ce6 (**C**), and Pheo (**D**) (hν+: irradiated, hν−: non irradiated).

The variation of the 3D-RI values among different kinds of analyzed samples is shown in Figure 5. The black boxes represent the distribution of the RI values before irradiation after 24 h of incubation with (see Figure 5B–D) or without photosensitizer (see Figure 5A) and the red boxes are 24 h after irradiation (hν+) or dark-control (hν−).

In case of non-irradiated samples without PS, it can be seen that the average RI value of bacteria cells present in this sample is slightly decreasing over time, which may be related to the beginning of the process of death of bacterial cells. Some population of bacteria cells started to die due to the lack of any nutrients in the NaCl solution, which led to the decrease of the averaged RI of these cells. For the nonirradiated samples with PS, the opposite tendency is observed. The increase in the averaged RI value may suggest further process of accumulation of PS over time, but not only, which will be discussed further in this section. However, in the case of irradiated samples with PS (after aPDT treatment), the decrease of the averaged RI values in time can be observed. This decrease related to the decrease in cellular density can be caused by the photodynamic inactivation of bacteria. This local decrease in intracellular density caused by aPDT may be related to ultrastructural changes in bacterial cells such as reactive oxygen species (ROS)/free radical-induced destruction of bacteria cell structures (DNA, cell wall, or ribosomes), protein and enzyme denaturation, inhibition of protein synthesis, or the aggregation of cytoplasmic macromolecules [14]. Therefore, it is possible to distinguish photoinactivated cells by their RI value [24].

**Figure 6:**
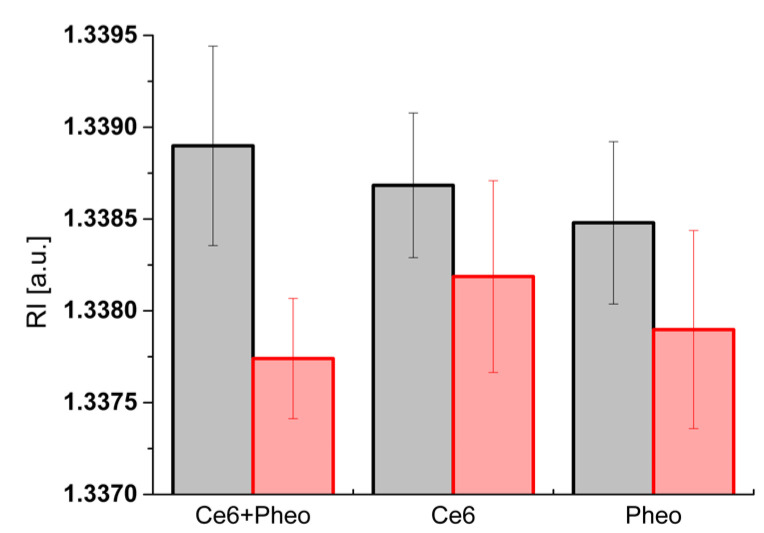
Column plot with error bar (standard deviation) showing the average RI values of cells before irradiation (black column) and 24 h after irradiation (red column).

Moreover, the change of RI values before and after irradiation can be used to evaluate the efficiency of bacteria photoinactivation. Comparison of the averaged RI values of bacteria cells (incubated with PS) before and 24 h after irradiation is shown in Figure 6. The greatest change in the averaged RI values was observed in cells before irradiation and 24 h after irradiation for Ce6 + Pheo. The differences for Pheo + Ce6, Ce6, and Pheo were 0.00116, 0.00050, and 0.00058, respectively. Only for Ce6 + Pheo do the error bars not overlap. The lower averaged RI value corresponds to the lower intracellular density related to the more effective photoinactivation of bacteria cells by this PS combination.

**Figure 7:**
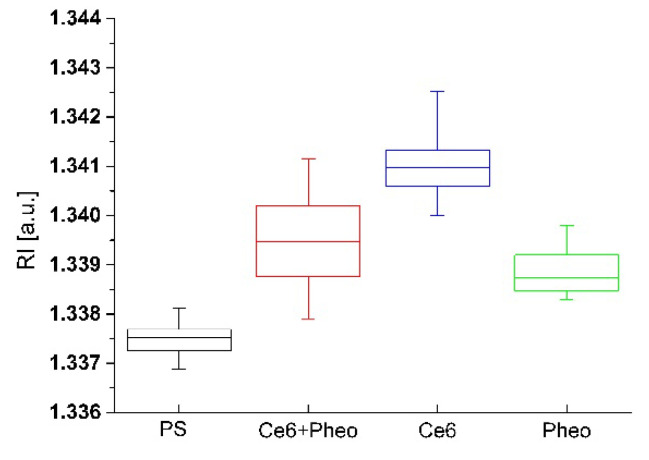
The changes of RI values of cells with accumulated photosensitizer and without PS after 48 h incubation in the case of non-irradiated samples.

Some additional observations occur after the analysis of the time-dependent differences of RI values between the samples. After 48 h of incubation with PS and without irradiation for all examined samples, an increase in the average RI of cells was observed. However, the greatest change was observed between the average RI value between 24 and 48 h of incubation for Ce6 and the smallest change was observed for Pheo (see Figure 7). 

**Figure 8:**
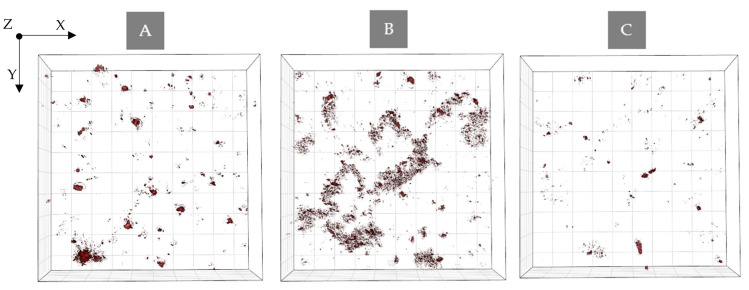
Digitally stained (based on the RI values) photosensitizer aggregates (**A**) Ce6 + Pheo, (**B**) Ce6, and (**C**) Pheo after 48 h incubation with PS.

This large change in RI in the case of Ce6 samples may be related not only to the more effective accumulation of PS within bacteria cells, as previously suggested, but also with the process of aggregation of PS in the case of the use of aqueous solvents. After 48 h of incubation with Ce6 a significant concentration of aggregated PS was observed near the bacterial cells. It was most visible in the case of the Ce6 group (see Figure 8A), while in the case of Ce6 + Pheo, and Pheo, this process was weaker since only large aggregates were present (see Figure 8B,C). On the basis of this observation, it can be concluded that PS aggregates located in the immediate surroundings of cells may overestimate the obtained averaged RI value. This effect can be limited in the case of Ce6 + Pheo, and Pheo, when large aggregates make it easier to eliminate them from the analysis. However, in the case of Ce6, which forms smaller aggregates, the limited lateral resolution of DHT can lead to artificial increase in the average RI value of cells, but not related to the accumulation of PS. 

**Figure 9:**
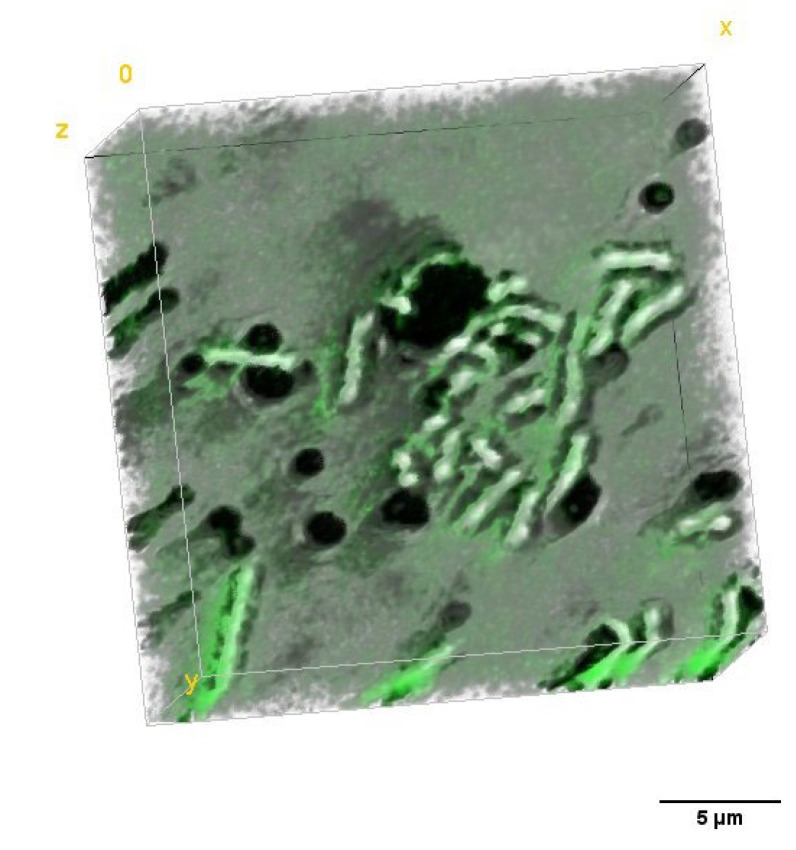
Ce6 aggregates localization performed by fluorescence confocal microscopy for *E. coli* cells after 48 h incubation with PS.

This effect can be explained by the mutual adhesion of cells and Ce6. The greater number of PS aggregates observed in the case of Ce6 may be caused by the mechanism of action of the efflux pump mechanism, which removes toxic substances from the cell into the surrounding medium and forms aggregates in the surroundings of the cells. Tetrapyrrolic compounds usually aggregate in aqueous media due to their low water solubility. This process is co-related with aggregation-caused quenching, the intensity of fluorescence, and the effectiveness of aPDT decreases. The aggregate states are caused by π–π stacking [30]. As can be seen in Figure 9, PS fluorescence was not observed in photosensitizer aggregates (non-emissive black structures). It should be noted that PS’s aggregation process can be limited by the use of different solvents.

To determine whether the observed changes of the averaged RI of cells caused by irradiation were statistically significant, a one-way ANOVA was performed. The normality assumption of the average RI values of cells was confirmed by the Kolmogorov–Smirnov test at 5% significance level. The ANOVA results are depicted in Table 2.

The estimated *p*-value for the F-statistic is significantly lower than the significance level (0.05) for the Ce6 + Pheo, Ce6, and Pheo groups, which means that the test rejected the null hypothesis that all the means of the group were equal. For PS group, the estimated *p*-value for the F-statistic is higher (0.119, see Table 2) than the significance level (0.05). The test cannot reject the null hypothesis that the means of two groups are equal. It indicates that irradiation has no stimulating effect on the growth of bacterial cells, because the significant change in the RI value after irradiation related to the cell division process (local increase in density) is not observed.

### 2.4. Analysis of the Dry Mass Density of Bacteria Cells

**Figure 10:**
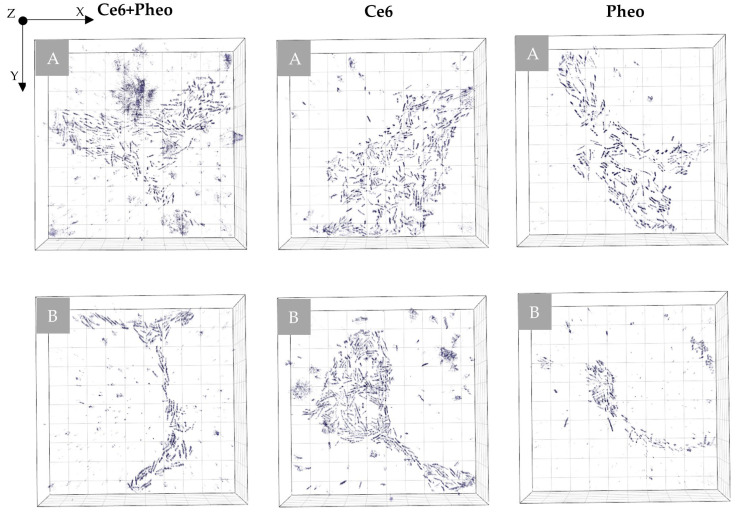
Representative digitally stained (based on the RI values) *E. coli* cells incubated with photosensitizers before (**A**) and 24 h after irradiation (**B**).

To determine the efficiency of the antimicrobial photodynamic therapy after photoexcitation of the cells incubated with PS, the 3D-RI distributions of *E. coli* biofilms that were reconstructed by DHT were used. The exemplary visualization of the digitally stained cells based on RI values of single cells are shown in Figure 10. The efficiency of aPDT is based on photoinactivation of cells by the laser-induced release of free radicals by accumulated PS. It is directly related to the dry mass density before and after photoexcitation. Based on the reconstructed 3D-RI tomograms by DHT and proposed algorithm for processing to obtain median RI value of cells, it was possible to determine the dry mass density of cells. 

**Figure 11:**
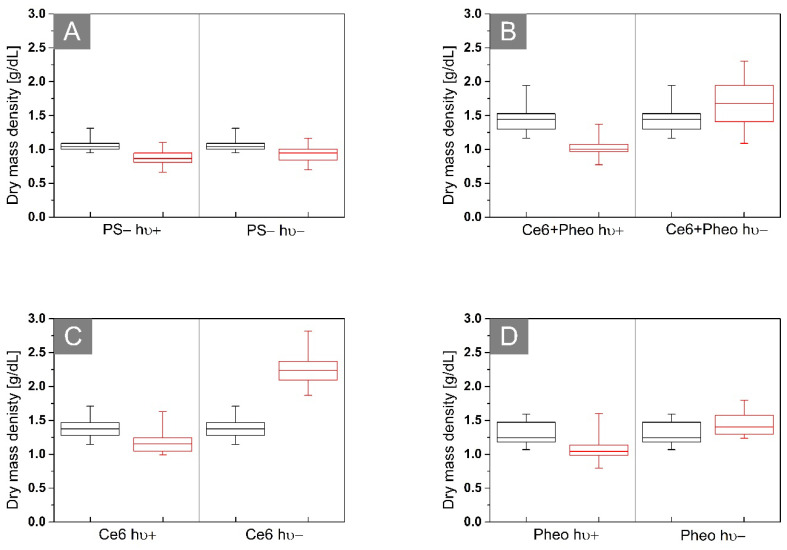
Representative box plots of dry mass density of biofilms of *E. coli* after incubation (**A**) without PS or with (**B**) Ce6 + Pheo, (**C**) Ce6, (**D**) Pheo, hν+: irradiated, hν−: not irradiated. The black boxes represent the distribution of RI values after 24 h, and the red boxes 48 h after the start of the experiment.

Photoinactivation of bacterial cells by PS leads to changes in the morphology of cellular structures. aPDT has an impact on the concentration of substances related to the chemical composition of these structures and, consequently, also the dry mass density of the cells. The cell survival rate can be confirmed by the dry mass test [31]. The results obtained from the dry mass density analysis are shown in Figure 11. The black boxes represent the density of the dry mass before irradiation (after 24 h of incubation with (see Figure 11B–D) or without photosensitizer (see Figure 11A)) and the red boxes are 24 h after irradiation (hν+) or dark-control (hν−). The average dry mass densities are shown in Table 3. 

Antimicrobial photodynamic therapy is accompanied by a change in the dry mass density of the cell [31], which is related to cell membrane damage and loss of cell integrity or to the DNA damage caused by the free radical. The decrease in dry mass density could be observed only in the case of the Ce6 hν+, Ce6 + Pheo hν+, and Pheo hν+ groups after laser light irradiation. Dark-control (hν−) groups are characterized by an increase in dry mass density between 24 h (black box) and 48 h (red box) of the experiment. It is related to processes such as cell division and further accumulation of PS within the bacteria. This demonstrates the lack of toxicity without irradiation. In the case of the PS group, it can be shown, as in the case of changes in bacterial RI, that irradiation has no stimulating effect on bacterial cells, which is manifested by the lack of significant differences in dry mass density between irradiated and non-irradiated cells (see Figure 11A). 

Based on the averaged dry mass density before and 24 h after irradiation, it was possible to evaluate the aPDT efficiency that was expressed as the difference between the dry mass density before irradiation and after irradiation, which is normalized, respectively, to the dry mass density before irradiation. For all analyzed samples with obtained aPDT, efficiency was equal to 29.45% for Ce6 + Pheo, 13.77% for Ce6, and 16.15% for Pheo. It can be seen that the aPDT efficiency was highest for the combination of PSs, and lowest for Pheo.

## 3. Materials and Methods

### 3.1. Bacterial Sample Preparation

In all experiments, the bacterial culture of *E. coli* (ATCC 25922) was used. *E. coli* was inoculated in tryptic soy agar (TSA) medium at 37 °C. The overnight culture stock was dissolved in NaCl (0.9% solution, Poch Basic, Gliwice, Poland), and the MacFarland scale (McF) was measured with a densitometer (DEN-1B, Biosan, Jozefow k Otwocka, Poland). A total of 0.5 McF was taken, which is approximately 1.5 × 10^8^ bacterial cells per mL. The bacteria culture was suspended in NaCl. For cell culture, the dish µ-Dish 35 low (IBIDI GmbH, Gräfelfing, Germany), was used. The refractive index of NaCl (0.9%) in which bacteria were suspended was equal to 1.335 and was measured with the Abbe refractometer (NAR-2T, minimum scale: 0.001, ATAGO Co. Ltd., Tokyo, Japan) at 20 °C.

Chlorin e6 (Ce6) [32], Pheophorbide a (Pheo) [33] (manufacturer Santa Cruz Biotechnology Inc., Dallas, TX, USA), and their combination were used as photosensitizers (PSs). Stock solutions of 0.84 × 10^−3^ M photosensitizer were prepared by dissolving the photosensitizer in NaCl. In this study, eight groups were examined: Ce6 hν+ (photoexcited), Ce6 hν− (dark-control), Ce6 + Pheo hν+ (photoexcited), Ce6 + Pheo hν− (dark-control), Pheo hν+ (photoexcited), Pheo hν− (dark-control), PS− hν+ (bright-control), and PS− hν− (dark-control). For each group, 24 samples were examined. The experiment was divided into three parts. On the first day, photosensitizing solutions were added to the parts of the samples. For samples with one photosensitizer, 100 μL of PS solution was added; when using a mixture of photosensitizers, 100 μL of each (Ce6 and Pheo) were added. In the next step, all samples were incubated for 24 h at 37 °C. Before imaging by digital holotomography and confocal microscopy, each sample was rinsed twice with 0.9% NaCl solution to remove PS from the medium. It was assessed whether the photosensitizer interacted with the cells. After assessment, part of the samples had been exposed to laser light (655 nm—wavelength used for aPDT). Subsequently, all samples were placed in a refrigerator for 24 h at 4 °C. The samples were rinsed again, and then the samples were imaged.

### 3.2. Spectrophotometric Measurements

All absorption spectra were recorded in the wavelength range 200–1100 nm by means of the AVA-Spec 3648 spectrophotometer (Avantes Inc., Apeldoorn, The Netherlands) equipped with a deuterium–halogen lamp (Avalight-DH-S-BAL, Avantes Inc., Apeldoorn, The Netherlands) as a light source. As the excitation source in the luminescence measurement, the continuous wave semiconductor laser λ = 405 nm (TOPGaN, Warsaw, Poland) was used. For spectroscopic studies, the PSs solutions in PBS were measured in standard UV cuvettes.

### 3.3. Irradiation Source for Bacteria Photoinactivation

The photoexcitation setup included a laser diode (λ = 655 nm) with an adjustable power control unit coupled to the optical fiber system (FC-655nm-1W-15070826, New Industries Optoelectronics Tech. Co., Ltd., Changchun, China). Exposure parameters have been determined experimentally. The most important element was that the sample (r = 7 mm) was exposed throughout the surface. The distance from the light source from the sample was set at 55 mm. The duration of exposure was set at 240 s; this is the longest possible time without heating the sample. The power density emitted by the laser was equal to 420 mW/cm^2^ and the energy density was equal to 12.5 J/cm^2^.

The energy density H and the power density E were set experimentally. The power density was measured by integrating a sphere photodiode power sensor (S142C, 350–1000 nm, 1 µW–5 W, Thorlabs, Newton, NJ, USA) and a compact power and energy meters console (PM100D, Thorlabs, Newton, NJ, USA). All exposures were continuously monitored by temperature measurement with a thermal imaging camera (FLIR E6, FLIR Systems, Inc., Wilsonville, OR, USA).

### 3.4. Confocal Laser Microscopy

Fluorescent or confocal microscopes are commonly used in studies of course, uptake of photosensitive compounds, and the effectiveness of photodynamic therapy; in addition, the morphology of bacterial cells is evaluated [19,34,35]. In comparison to digital holotomography (DHT), these microscopes are much more expensive because it is necessary to use fluorescence markers/probes and it is much more time-consuming since the process of scanning the sample takes longer. 

The images of bacterial cells were collected on a Leica TCS SPE confocal laser microscope (Leica, Wetzlar, Germany) using a 63× high-numerical-aperture oil-immersion objective. This microscope was working in the fluorescence and differential interference contrast (DIC) modes. Furthermore, X–Z and Y–Z cross sections were recovered to obtain 3D information indicating PS uptake. The image size was set to 512 × 512 pixels. For excitation of the PS laser line at 405 nm, it was operated at 10% of the maximum power.

In the first stage of the study, the bacterial samples were examined by confocal microscopy to confirm the lack of the autofluorescence of bacterial biofilm. After photoexcitation of the PS by laser light with the wavelength equal to 405 nm (corresponding to the PDD absorption bands of both photosensitizers), confocal microscopic images indicating the cell penetration by PS were registered. 

### 3.5. Digital Holographic Tomography and RI Data Processing

In this study, a DHT was used for examination of the accumulation of photosensitizers inside the bacteria cells and changes in cell density related to the aPDT treatment, since, contrary to the fluorescence confocal microscopy, it allows for a quick and non-invasive process of scanning a sample, and the obtained results in the form of a spatial distribution of the refractive index may give equally interesting results. 

A digital holotomograph (3D Cell Explorer, Nanolive, Ecublens, Switzerland) using a dry microscope objective (60×, numerical aperture NA = 0.8, Nikon) was used to visualize the spatial distribution of the refractive index (RI) in the form of 3D-RI tomograms. During the scanning of the sample, multiple two-dimensional RI measurements are made, which, when numerically reconstructed, give a 3D distribution of the RI examined sample. 

Numerically reconstructed 3D-RI tomograms were imported from STEVE software (version 1.6.3496, Nanolive, Ecublens, Switzerland) to MATLAB^®^ software (version R2021b, MathWorks, Natick, MA, USA). To obtain the RI values of the bacteria cells, a single 2D-RI tomogram was selected, representing the single slice of 3D-RI tomogram for which the best contrast of the examined cells was obtained. The applied algorithm performed median filtering to reduce artifacts that were present in the selected 2D-RI tomograms. In the next step, a contour mask was created that was based on the specified polygonal region of interest. This region was matched by appropriately specifying a threshold for the RI values, which exceeded the RI of the medium. The contour mask was fitted on the original data (a series of 2D-RI tomograms), enabling automatic distinguishing of the regions that were occupied by the cells and to directly determine the RI values of each cell that is present in the sample. As a result, the RI values of the pixels of the regions that were occupied by cells were obtained. A median RI value was determined for each tomogram. To obtain a representative set of data for this analysis, 20 tomograms (3D-RI) of each group were examined. Then, the data were analyzed.

RI is one of the physical parameters that can be correlated with biophysical parameters such as dry mass density. Significant changes in protein composition and other cell components can be detected by changes in the RI values. This could be related to abnormality and dysfunction, showing intrinsic dynamics in cells [36]. 

The dry mass density of the cell ($M_{dry}$) can be expressed as [37,38]:

(1) $$M_{dry}=\left(\frac{RI}{RI_{NaCl}}-1\right)\cdot \frac{1}{k}$$

where RI is the median of the refractive index of the cells, $RI_{NaCl}$ is *RI* of the environment (1.335), and k is the absorption constant; for material that has no specific light absorbance characteristic, k = 0.002.

### 3.6. Statistical Analysis

Statistical analyses were performed using Origin 2021 (OriginLab Corporation, Northampton, MA, USA). The significance of RI value was assessed by one-way analysis of variance (ANOVA). A value of *p* < 0.05 was considered as statistically significant.

## 4. Conclusions

A combined therapy becomes the most common strategy in modern treatment. Its greatest advantages are using different pathways, achieving higher efficacy and minimizing side effects. Furthermore, the possibility of developing resistance is reduced. This strategy allows to obtain the additive or synergistic effect of treatment using smaller doses of each substance. The use of two photosensitizers in a single treatment is considered to be a basic aPDT combination. Other strategies are based on combinations of aPDT with antibiotics, other antimicrobial compounds, or physical treatments (sonodynamic therapy and electrochemotherapy) [10].

The results obtained in this in vitro study show that the use of the functionalized combination of two photosensitizers, Ce6 and Pheo, in which Pheo acts as an efflux pomp inhibitor, enables the penetration of increase of the *E. coli* (Gram-negative bacteria) cells by PSs, which is significantly higher than in case of the use of each of PS individually. The increase in PS accumulation was indicated by the increase of the RI values of individual cells related to the increase in cell density in the presence of PSs, which was confirmed by the additional confocal fluorescence microscopy. Moreover, the decrease of the RI values of bacteria cells after aPDT treatment can indicate the efficiency of *E. coli* inactivation related to the decrease of the cell density and dry mass density obtained from RI data, which may be correlated with the loss of the integrity of cells membrane, increase in membrane permeability, or DNA damage characteristic for aPDT. The results suggest that when a functionalized combination of PSs was used, the efficacy of the photodynamic effect was significantly higher than when each of the sensitizers was used alone. Furthermore, the use of the developed functional combination of PSs can contribute to a more efficient photoinactivation of Gram-negative bacteria cells, which have a lower permeability to single anionic and neutral photosensitizers compared to those of Gram-positive bacteria cells. However, the results indicate the PS aggregation in the used NaCl suspension, so future works should focus on the elimination of this effect by the use of a different solvent that can enhance the photodynamic inactivation of Gram-negative bacteria.

Digital holotomography (DHT), using the refractive index as an imaging contrast for 3D label-free live bacteria cell imaging and providing quantitative information about biophysical properties of the specimen, can be used in complex examination of the single-cell–photosensitizer interaction and characterization of the efficiency of aPDT as a low-cost, label-free, nondestructive alternative to commonly used techniques such as confocal microscopy.

## Figures and Tables

**Table 1:** The results of the ANOVA, where each of the 4 groups (Ce6 + Pheo, Ce6, Pheo, PS−) represent the set of average RI of *E. coli* cells after 24 h incubation.

Source of Variability	SS ^1^	df ^1^	MS ^1^	F ^1^	Prob > F
Group (between)	1.254 × 10^−5^	3	4.180 × 10^−6^	23.586	**<<0.05**
Error (within)	1.347 × 10^−5^	76	1.772 × 10^−7^		
Total	2.601 × 10^−5^	79			

^1^ SS—is a sum of squares due to each source, df—degree of freedom associated with each source, MS—mean squares for each source, F—F-statistics, Prob > F—*p*-value which is the probability that F-statistic can take a value larger than computed F-statistic value.

**Table 2:** The results of the ANOVA, where each from 2 groups represent the set of average RI of *E. coli* cells after irradiation/bright-control (hν+) and dark-control (hν−) samples.

**PS−**					
**Source of variability**	**SS ^1^**	**df ^1^**	**MS ^1^**	**F ^1^**	**Prob > F**
Group (between)	2.337 × 10^−7^	1	2.337 × 10^−7^	2.538	**0.119**
Error (within)	3.499 × 10^−6^	38	9.207 × 10^−8^		
Total	3.732 × 10^−6^	39			
**Ce6 + Pheo**					
**Source of variability**	**SS ^1^**	**df ^1^**	**MS ^1^**	**F ^1^**	**Prob > F**
Group (between)	2.903 × 10^−5^	1	2.903 × 10^−5^	57.539	**<<0.05**
Error (within)	1.917 × 10^−5^	38	5.045 × 10^−7^		
Total	4.820 × 10^−5^	39			
**Ce6**					
**Source of variability**	**SS ^1^**	**df ^1^**	**MS ^1^**	**F ^1^**	**Prob > F**
Group (between)	8.265 × 10^−5^	1	8.265 × 10^−5^	249.346	**<<0.05**
Error (within)	1.260 × 10^−5^	38	3.315 × 10^−7^		
Total	9.525 × 10^−5^	39			
**Pheo**					
**Source of variability**	**SS ^1^**	**df ^1^**	**MS ^1^**	**F ^1^**	**Prob > F**
Group (between)	9.604 × 10^−6^	1	9.604 × 10^−6^	36.623	**<<0.05**
Error (within)	9.965 × 10^−6^	38	2.622 × 10^−7^		
Total	1.957 × 10^−5^	39			

^1^ SS—is a sum of squares due to each source, df—degree of freedom associated with each source, MS—mean squares for each source, F—F-statistics, Prob > F—*p*-value which is the probability that F-statistic can take a value larger than computed F-statistic value.

**Table 3:** The averaged dry mass density with standard deviation before and after irradiation for all analyzed photosensitizers.

Photosensitizers	Ce6 + Pheo		Ce6		Pheo	
Groups	Before irradiation	After irradiation	Before irradiation	After irradiation	Before irradiation	After irradiation
Mean dry mass density (g/dL)	1.46 ± 0.20	1.03 ± 0.12	1.38 ± 0.15	1.19 ± 0.20	1.30 ± 0.17	1.09 ± 0.20

## Data Availability

All data can be obtained from authors on reasonable request.
